# Recurrent cerebrospinal fluid herpesvirus PCR positivity: a retrospective analysis of inter-positive intervals

**DOI:** 10.1099/jmm.0.002222

**Published:** 2026-11-11

**Authors:** Ji Yeon Kim, Bo Kyeung Jung, Jae-Sik Jeon, Jae Kyung Kim

**Affiliations:** 1 Department of Biomedical Laboratory Science, College of Health Sciences, Dankook University, Cheonan-si 31116, Republic of Korea; 2 Department of Laboratory Medicine, Dankook University Hospital, Cheonan-si 31116, Republic of Korea

**Keywords:** cerebrospinal fluid, herpesvirus, HHV-6, inter-positive interval, molecular diagnostics, recurrent positivity

## Abstract

**Introduction.** Herpes simplex virus types 1 and 2 (HSV-1 and HSV-2) and varicella-zoster virus (VZV) are established causes of central nervous system infection, whereas human herpesvirus 6 (HHV-6) DNA may be detected in more heterogeneous contexts. However, longitudinal patterns of recurrent cerebrospinal fluid (CSF) PCR positivity remain poorly characterized.

**Hypothesis/Gap statement.** When the same herpesvirus is detected in CSF on different dates, the meaning of the interval between positive results is uncertain. Its observed distribution may also depend on retesting opportunities and changes in testing practice.

**Aim.** To describe inter-positive intervals across five herpesvirus targets and assess how their interpretation changes with operational cut-offs and calendar-period testing patterns.

**Methodology.** We retrospectively analysed qualitative CSF PCR results for five herpesviruses from 16 March 2010 to 30 March 2026. Recurrent positivity required at least two distinct positive dates per patient–virus pair. First-to-last intervals were classified at 30 days, with exploratory cut-offs of 14, 60 and 90 days. Calendar-period analyses used FilmArray Meningitis/Encephalitis panel introduction on 10 November 2020 as the boundary and period-specific positive pairs as denominators.

**Results.** Among 19,959 interpretable target-level results (994 positive), 33 recurrent pairs were identified: HHV-6 (18), VZV (7), HSV-1 (5), HSV-2 (2) and cytomegalovirus (1). The median first-to-last inter-positive interval was 11 days (interquartile range, 4–185; range, 1–864). Nineteen intervals were <30 days and 14 were ≥30 days; HHV-6 accounted for 10 longer pairs, all pre-implementation, limiting target-specific interpretation. The HHV-6 versus non-HHV-6 comparison was not significant (10/18 versus 4/15; two-sided Fisher’s exact *P*=0.158). Within-period recurrent positivity was 24/771 (3.1%) in the pre-implementation calendar period and 8/176 (4.5%) in the post-implementation period; corresponding HHV-6 recurrent positivity was 17/302 (5.6%) and 1/84 (1.2%).

**Conclusion.** Recurrent CSF herpesvirus PCR positivity showed heterogeneous target composition and inter-positive intervals. These data cannot distinguish clustered retesting, persistence, reactivation or chromosomal integration. Interpretation requires prior testing history and linked clinical or quantitative virological data.

**Impact Statement**

Recurrent cerebrospinal fluid (CSF) herpesvirus PCR positivity can have several explanations. In this 16 year laboratory dataset, recurrent patient–virus pairs showed heterogeneous inter-positive intervals and target composition, with human herpesvirus 6 (HHV-6) contributing to most pairs separated by 30 days or more. Seventeen of the eighteen recurrent HHV-6 pairs occurred pre-implementation; assay and workflow differences, unequal observation periods and other calendar-period effects may, therefore, influence this distribution. Retesting opportunity and period-specific denominators provide context for the observed recurrent positivity; these proportions do not estimate clinical recurrence risk. Inter-positive interval may, therefore, provide contextual laboratory history, but it should not be used as a surrogate for persistence, reactivation or chromosomal integration. Clinical course, treatment exposure and quantitative or integration-focused testing, where appropriate, are needed to assess the clinical meaning of repeated detection.

## Data Summary

 Individual-level laboratory information system data are not publicly available because they contain potentially re-identifiable clinical laboratory metadata, including testing dates, age, sex, pathogen targets and repeat-testing patterns and are subject to institutional data-governance and ethics restrictions. Aggregate data supporting the tables and figures are provided within the article and supplementary material. The Supplementary Material additionally reports consecutive-positive intervals for eight trajectories using study labels without source identifiers or calendar dates. Analysis code and aggregate outputs used for verification are retained as internal validation files and are not included in the formal Supplementary Material; regeneration of individual-level analyses requires the controlled institutional source workbook. No external datasets, accession numbers or repository identifiers apply to this study.

## Introduction

Syndromic multiplex PCR panels are routinely used for the rapid evaluation of suspected central nervous system (CNS) infections, allowing simultaneous detection of bacterial, viral and fungal pathogens directly from cerebrospinal fluid (CSF) [1, 2]. Although syndromic testing has shortened diagnostic turnaround, qualitative all-or-none output can create interpretive challenges when the same target is detected repeatedly in one patient [3]. Herpesviruses are particularly relevant because they can establish latency and can be detected in clinically heterogeneous settings [4]. Herpes simplex virus type 1 (HSV-1) is classically associated with encephalitis, herpes simplex virus type 2 (HSV-2) with aseptic and recurrent lymphocytic meningitis, varicella-zoster virus (VZV) with meningitis or encephalitis and human herpesvirus 6 (HHV-6) with CNS disease, particularly in selected immunocompromised populations; inherited chromosomally integrated HHV-6 (iciHHV-6) can also produce DNA detection independent of active viral replication [4–12].

Repeated CSF herpesvirus PCR positivity may, therefore, arise during continued diagnostic evaluation, persistent or incidental DNA detection, recurrent viral activity or – specifically for HHV-6 – chromosomal integration [6–12]. Recent clinical virology studies have emphasized that HHV-6 DNA in CSF can represent either clinically relevant infection or a bystander signal, and that qualitative detection alone should not be equated with active CNS disease [13–15].

HSV infection is common worldwide, with global estimates indicating billions of HSV-1 infections and hundreds of millions of HSV-2 infections [16]. In Korea, HSV-1 and HSV-2 have also been identified among PCR-confirmed herpesvirus CNS infections in adults evaluated for aseptic meningitis or encephalitis [17]. When HSV encephalitis is suspected, intravenous acyclovir should be started promptly and is generally recommended for 14–21 days [18]. Relapse after treatment can occur, and recurrent HSV-2 meningitis may occur over longer periods [11, 12, 18]; however, no single post-treatment interval can distinguish persistence, relapse or reactivation from a qualitative CSF PCR result alone.

Clinically adjudicated recurrent lymphocytic meningitis cohorts have documented repeated HSV PCR positivity across separate disease episodes; in one series, HSV DNA was detected in more than one episode in 6 of 11 patients with at least one PCR-positive episode [19]. Broader syndromic-panel studies have not generally treated repeat positivity as the primary longitudinal outcome; in a recent FilmArray ME series focused on bacterial and fungal meningitis, 17 consecutively positive panels were identified and excluded from the statistical analysis [20]. Longitudinal laboratory patterns of repeated positivity across herpesvirus targets and changing testing workflows, therefore, remain less well described. What can the interval between repeated positive CSF herpesvirus PCR results tell us when clinical episode boundaries and the reasons for retesting are unknown? We characterized recurrent patient–virus pairs across five herpesvirus targets, examined their intervals using operational cut-offs and assessed retesting opportunity and calendar-period patterns around the 2020 workflow change.

## Methods

### Study design and setting

This retrospective observational study used laboratory information system (LIS) data from Dankook University Hospital, a tertiary-care hospital in Cheonan, Republic of Korea. Reporting followed the Strengthening the Reporting of Observational Studies in Epidemiology statement and the REporting of studies Conducted using Observational Routinely-collected health Data extension [21, 22]. The study was approved by the Institutional Review Board of Dankook University Hospital (DKUH IRB 2026-05-020; approved 11 June 2026; expedited review). The de-identified dataset was extracted on 12 June 2026. The requirement for informed consent was waived because the study used retrospectively collected, de-identified laboratory data.

### Specimen processing and PCR testing

CSF specimens were generally tested within 24 h of receipt by the laboratory. Specimens awaiting molecular testing were stored at 2–8 °C until analysis. During use of the BioFire FilmArray Meningitis/Encephalitis (ME) panel, specimen handling and testing were performed according to the manufacturer’s instructions.

Before 10 November 2020, CSF herpesvirus testing was performed using the Seeplex^®^ Meningitis-V1 ACE Detection kit (Seegene Inc., Seoul, Republic of Korea), a commercial conventional multiplex PCR assay with electrophoretic detection. The assay targeted HSV-1, HSV-2, VZV, Epstein–Barr virus (EBV), cytomegalovirus (CMV) and HHV-6. The present recurrent-positivity analysis included HHV-6, HSV-1, HSV-2, VZV and CMV when an interpretable result was available. EBV results were not retained in the archived longitudinal extraction used to construct the analytic dataset and were, therefore, excluded from the recurrent-positivity analysis.

On 10 November 2020, the laboratory implemented the BioFire FilmArray ME panel (BioFire Diagnostics, Salt Lake City, UT, USA) as part of routine workflow revision. Selected-target testing continued for some orders after implementation, and row-level instrument identity was not consistently retained in the retrospective analytic extract. Accordingly, calendar periods before and after 10 November 2020 were treated as implementation periods rather than mutually exclusive assay-platform groups. CMV was included only when reported because its longitudinal availability differed from that of the four principal herpesvirus targets. The performance of the FilmArray ME Panel and the interpretive challenges of HHV-6 detection have been described previously [1, 2, 23].

### Clinical data availability

The de-identified LIS dataset was not comprehensively linked to inpatient versus outpatient status, ordering-clinician identity, the clinical indication for repeat testing, symptom trajectory, final neurological diagnosis, antiviral treatment or changes in therapy, treatment adherence, antiviral resistance, immune status, CSF cell counts or protein, neuroimaging findings or quantitative viral-load or cycle-threshold (Ct) data. These variables, therefore, could not be reconstructed reliably. Recurrent patient–virus pairs were consequently not adjudicated as persistence, reactivation, recurrent infection or chromosomal integration; these mechanisms are discussed only as possible biological contexts for recurrent qualitative detection.

### Data extraction and definitions

All CSF PCR results issued between 16 March 2010 and 30 March 2026 were extracted from the LIS. Records outside these dates, including archived CMV results from 2006 to 2009, were excluded. Tests with non-binary or uninterpretable results were excluded. A testing episode was defined by a unique patient identifier and order date. A patient was classified as repeatedly tested when testing episodes occurred on two or more distinct dates. At the patient–virus level, a same-target retested pair had an interpretable result for the same virus on two or more distinct dates. Recurrent positivity was defined as two or more positive PCR results for the same virus in the same patient on different order dates. The inter-positive interval was calculated as the number of days between the first and last positive result for each recurrent patient–virus pair. Same-day patient–virus records were consolidated using a positive-wins rule (positive if any same-day result was positive) before identifying distinct positive dates. For pairs with more than two positive dates, consecutive inter-positive intervals were additionally calculated between adjacent positive dates in chronological order, irrespective of intervening negative tests; the first-to-last interval remained the primary measure.

The primary descriptive classification used intervals of <30 and ≥30 days. The 30-day value was an operational threshold selected to distinguish closely clustered repeat positivity from positivity separated by a longer period; it was not considered a biological cut-off or a validated episode boundary. Robustness was examined using alternative cut-offs of 14, 60 and 90 days.

For the implementation-period sensitivity analysis, recurrent pairs were classified as pre-implementation when both the first and last positive dates occurred before 10 November 2020, post-implementation when both occurred on or after that date, and transition-spanning when the first positive date preceded implementation and the last positive date occurred on or after implementation. Raw interpretable and positive target-level results were summarized by calendar period before same-day consolidation. The crude recurrent-pair count per 1,000 interpretable target-level results was calculated using recurrent trajectories occurring wholly within the corresponding period; this was treated as a volume-normalized descriptive index, not a patient-level disease-recurrence probability. For positive-pair denominators, same-day records for each patient–virus pair were consolidated to one date-level result using a positive-wins rule. Within each period, a positive pair was a unique patient–virus combination with at least one positive result; a positive pair was classified as subsequently retested when any later same-target result occurred in the same period and as recurrent-positive within the period when two or more distinct positive dates occurred in that period. The transition-spanning trajectory was reported separately and excluded from period-specific recurrent numerators. Positive-pair denominators retained all pairs with an eligible positive result in the corresponding period, including a pair also observed in the other period. Because selected-target testing continued after FilmArray ME introduction, these calendar-based analyses assessed temporal concentration rather than analytical equivalence between assay platforms.

### Statistical analysis

Categorical variables were summarized as counts and percentages. First-to-last inter-positive intervals were summarized using the median, interquartile range (IQR) and range; quartiles were calculated by linear interpolation. Recurrent patient–virus pairs were tabulated by viral target and inter-positive interval class. The proportion of pairs with intervals ≥30 days was compared between HHV-6 and non-HHV-6 herpesvirus targets using a two-sided Fisher’s exact test as an exploratory analysis. Because the number of recurrent pairs was small, inferential comparisons were interpreted cautiously and multivariable modelling was not performed. The classification was recomputed at alternative cut-offs of 14, 60 and 90 days; *P*-values from these analyses were considered exploratory and were not adjusted for multiple testing. An isolated nominal *P*-value below 0.05 across these exploratory thresholds was not treated as evidence of a statistically supported target association; interpretation focused on the descriptive interval distribution.

The implementation-period analysis was descriptive. We summarized observation duration, raw interpretable and positive target-level results, interpretable patient–virus–date results after same-day consolidation, positive patient–virus pairs, subsequent same-target retesting and within-period recurrent positivity, including corresponding HHV-6-specific denominators and target-level positivity rates. No formal between-period hypothesis test was planned because of the small number of recurrent pairs, unequal follow-up opportunity, and the non-exclusive relationship between calendar period and assay platform. Analyses used Python 3.12.14 with pandas 2.2.3, NumPy 2.3.5, SciPy 1.17.0 and Matplotlib 3.10.8. ChatGPT (OpenAI; web application, accessed 9 and 29 September 2026) and Claude (Anthropic; web application, accessed 12 June 2026) were used to assist with development and verification of deterministic data-processing and analysis code. The authors reviewed the input-variable definitions, code logic and aggregate outputs against the controlled source workbook. All reported analyses were executed using the specified Python libraries, and scientific interpretation remained the responsibility of the authors. Randomization and blinding were not applicable and no a priori sample-size calculation was performed because all eligible LIS records during the study period were included. Interval and denominator calculations were checked using a separate implementation of the same definitions.

### Data overlap transparency statement

This study used the same institutional CSF molecular-testing database as a previously published analysis by the author group [24]. That report examined age-specific viral detection, denominator choice and workflow effects across a broader set of viral targets and included a first-positive-per-patient sensitivity analysis. The present study addresses a different question by restricting the analysis to five herpesvirus targets and characterizing recurrent patient–virus pairs and their inter-positive intervals. The studies, therefore, share source records but differ in target scope, analytic unit, outcomes and interpretation. No text, table or figure is reused.

## Results

### Recurrent positive patient–virus pairs

Among 4,272 patients contributing 4,963 testing episodes, 492 (11.5%) underwent CSF herpesvirus PCR testing on two or more distinct dates. At the patient–virus level, 1,934 pairs had the same target tested on at least two dates; 33 (1.7%) had positive results on two or more dates and constituted the recurrent patient–virus pairs analysed here. These 33 pairs represented 33 unique patients and no patient contributed more than one recurrent viral target.

The analytic dataset contained 19,959 interpretable herpesvirus target-level results, including 994 positive results. HHV-6 was the most frequent recurrent target (18 pairs), followed by VZV (7), HSV-1 (5), HSV-2 (2) and CMV (1). The distribution of recurrent pairs by viral target and inter-positive interval is summarized in Fig. 1.

**Fig. 1.**
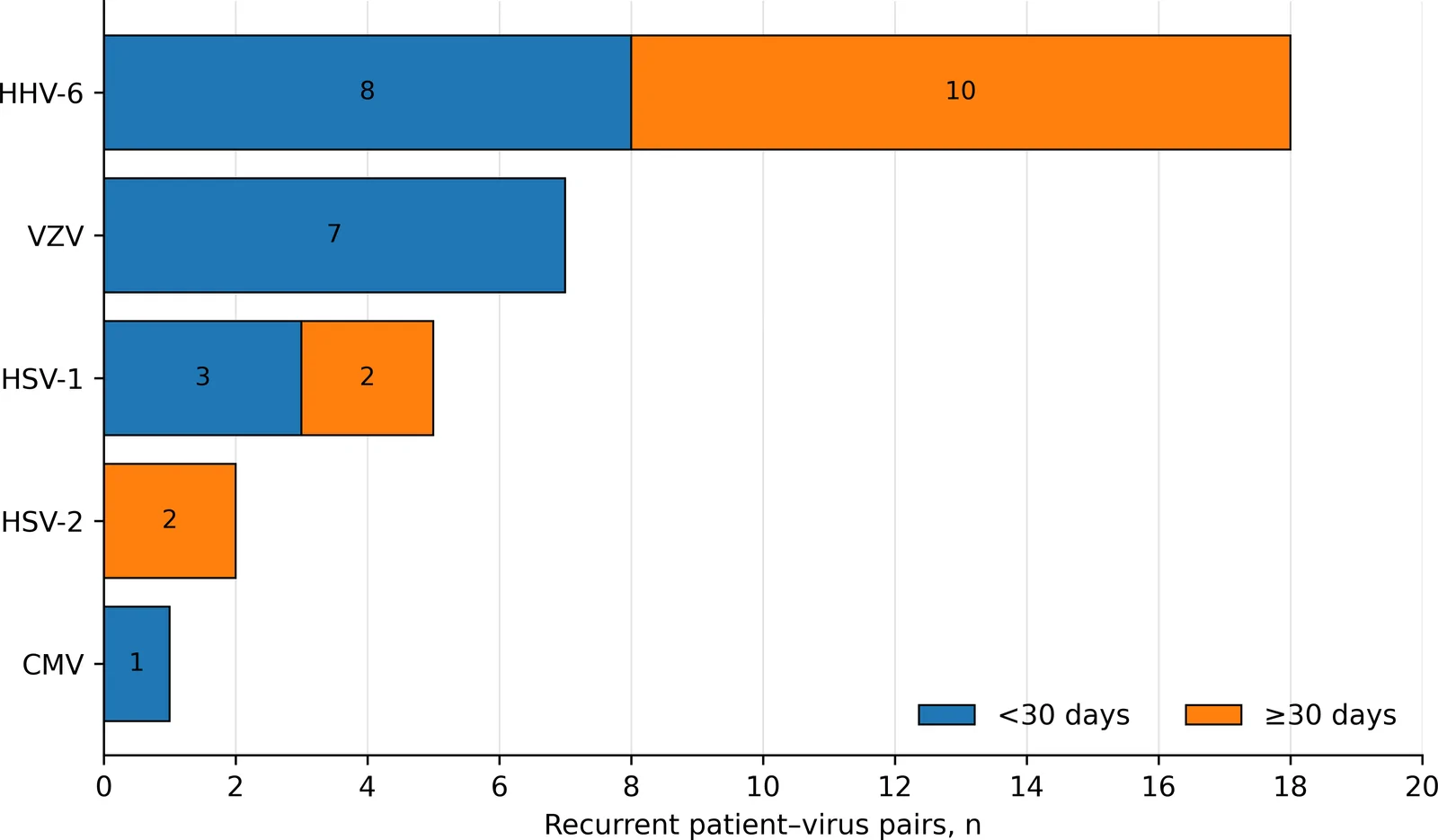
Target-specific distribution of recurrent CSF herpesvirus PCR positivity by inter-positive interval. Intervals <30 and ≥30 days are operational descriptive categories. Target-specific distributions should be interpreted cautiously because several viral targets had small recurrent-pair counts. All 10 HHV-6 pairs with intervals ≥30 days occurred in the pre-implementation period; calendar-period differences limit target-specific interpretation*.*

### Inter-positive interval distribution

The median first-to-last inter-positive interval was 11 days (IQR, 4–185; range, 1–864). Nineteen of the 33 recurrent pairs (57.6%) had inter-positive intervals <30 days, whereas 14 (42.4%) had intervals ≥30 days. These categories describe the timing of repeated qualitative positivity only. Admission-level episode boundaries and the clinical reasons for repeat sampling were not available, so <30 day pairs could not be confirmed as belonging to a single clinical episode, and ≥30 day pairs could not be interpreted as confirmed reactivation or recurrent infection. HHV-6 accounted for 10 of the 14 pairs with intervals ≥30 days (71.4%). All ten of these longer-interval HHV-6 pairs occurred pre-implementation, so this distribution may reflect calendar-period differences rather than a reproducible target-specific pattern. Eight of the thirty-three pairs (24.2%; five HHV-6, two HSV-1 and one HSV-2) had more than two distinct positive dates: four had three, three had four and one had seven. The remaining 25 pairs each had two positive dates.

### Recurrent positivity by herpesvirus target

Target-specific interval distributions were heterogeneous. Intervals ≥30 days occurred in 10 of 18 HHV-6 pairs (55.6%) and 4 of 15 non-HHV-6 pairs (26.7%). This exploratory comparison did not reach statistical significance (two-sided Fisher’s exact *P*=0.158). All seven recurrent VZV pairs had intervals <30 days, whereas both HSV-2 pairs had intervals ≥30 days. However, these small target-specific counts are descriptive and do not establish distinct biological mechanisms.

In sensitivity analyses using 14-, 60- and 90 day cut-offs, HHV-6 remained the most frequent target among pairs classified into the longer-interval category, accounting for 72.7–76.9% of such pairs. All recurrent VZV pairs remained in the shorter-interval category at every cut-off examined. The nominal *P*-value for HHV-6 versus non-HHV-6 varied from 0.037 at 14 days to 0.266 at 90 days, showing dependence on the selected operational cut-off (Tables S1a and S1b, available in the online Supplementary Material). Table S2 provides the continuous interval summaries, and Fig. S1 shows all 33 observed intervals by viral target. The isolated nominal *P*=0.037 at 14 days, among multiple unadjusted exploratory comparisons, does not establish a statistically supported target association.

### Distribution relative to the 2020 implementation transition

Of the 33 recurrent trajectories, 24 occurred entirely before 10 November 2020, 8 occurred entirely on or after that date and 1 spanned the implementation boundary. The two calendar periods differed in duration and testing volume. Before same-day consolidation, the 10.66 year pre-implementation period contained 13,149 interpretable target-level results, including 809 positive results (6.2%), whereas the 5.39 year post-implementation period contained 6,810 interpretable results, including 185 positive results (2.7%). The corresponding crude counts of within-period recurrent-positive trajectories were 1.83 and 1.17 per 1,000 interpretable target-level results, respectively (Table 1A). Table S3 summarizes these trajectories by period and interval class.

**Table 1.** Denominator-aware comparison by calendar period relative to FilmArray ME implementation

**A. Target-level testing volume and crude recurrent-pair frequency**				
**Calendar period**	**Interpretable target-level results, *n***	**Positive target-level results, *n* (%)**	**Recurrent pairs per 1,000 interpretable results**	
Pre (16 March 2010–9 November 2020; 10.66 years)	13,149	809 (6.2)	1.83	
Post (10 November 2020–30 March 2026; 5.39 years)	6,810	185 (2.7)	1.17	
**B. Positive-pair follow-up and recurrent positivity**				
**Implementation period**	**Positive pairs, *n***	**Retested pairs, *n* (%)**	**Recurrent-positive pairs, *n* (%)**	**Recurrent-positive HHV-6, *n*/*N* (%)**
Pre (16 March 2010–9 November 2020; 10.66 years)	771	57 (7.4)	24 (3.1)	17/302 (5.6)
Post (10 November 2020–30 March 2026; 5.39 years)	176	23 (13.1)	8 (4.5)	1/84 (1.2)
Transition-spanning	–	–	1	0
All recurrent trajectories	–	–	33	18

After same-day consolidation, the periods contained 13,091 and 6,765 interpretable patient–virus–date results. Among 771 positive patient–virus pairs in the pre-implementation period, 57 (7.4%) underwent a subsequent same-target test and 24 (3.1%) met the within-period recurrent-positivity definition. The corresponding post-implementation values were 23/176 (13.1%) retested and 8/176 (4.5%) recurrent-positive (Table 1B). Among subsequently retested positive pairs, within-period recurrent positivity was 24/57 (42.1%) before and 8/23 (34.8%) after implementation (Table S4). These are descriptive proportions within selectively retested subgroups, not estimates of recurrence risk. The transition-spanning HSV-2 pair had one positive date in each period and, therefore, contributed to both positive-pair denominators but neither within-period recurrent numerator; 24+8+1 accounts for all 33 recurrent pairs.

For HHV-6 specifically, target-level positivity was 329/3,329 (9.9%) in the pre-implementation period and 85/1,559 (5.5%) in the post-implementation period; within-period recurrent positivity was observed in 17/302 (5.6%) and 1/84 (1.2%) period-specific HHV-6-positive pairs, respectively. The numbers of recurrent pairs with intervals <30 and ≥30 days were 12/12 in the pre-implementation period, 7/1 in the post-implementation period and 0/1 among transition-spanning pairs (Table S3). Detailed period-specific denominators are provided in Table S4. Seventeen of the 18 recurrent HHV-6 pairs were pre-implementation and one was post-implementation; differences in assay mix, observation duration and calendar-period context warrant caution when interpreting target-specific differences. Table S5 reports consecutive-positive gaps and first-to-last spans for the eight pairs with more than two positive dates.

Implementation periods are calendar-based and do not represent mutually exclusive assay-platform groups. Panel A uses raw target-level results before same-day consolidation; its recurrent-pair frequency is a volume-normalized descriptive index, not a patient-level disease-recurrence probability and excludes the transition-spanning trajectory. In Panel B, same-day records were consolidated using a positive-wins rule. Positive pairs are unique patient–virus combinations with at least one positive result in the stated period; retested pairs had any later same-target result in that period; and recurrent-positive pairs had at least two positive dates in that period. Percentages use the corresponding period-specific positive-pair denominator. The transition-spanning pair is excluded from period-specific recurrent numerators but remains eligible for each period’s positive-pair denominator. The transition-spanning pair and all-trajectory totals are shown without period-specific percentages. For recurrent-positive HHV-6, *N* is the number of HHV-6-positive pairs. Period-specific pair denominators are not additive because a patient–virus pair may appear in both periods.

## Discussion

### Principal findings

Inter-positive intervals described the timing of repeated laboratory detection, but the available data could not establish clinical episode boundaries or the underlying biological mechanisms. In this 16-year study, we identified 33 recurrent CSF herpesvirus PCR-positive patient–virus pairs across five viral targets. HHV-6 accounted for 10 of the 14 pairs with intervals ≥30 days. All 10 HHV-6 pairs occurred before implementation, limiting target-specific interpretation. The primary 30-day comparison was not statistically significant (*P*=0.158), and the exploratory *P*-values varied with the operational cut-off. The isolated nominal *P*=0.037 at 14 days does not provide confirmatory evidence given the small sample and multiple unadjusted comparisons.

Previous studies have documented herpesvirus DNA in CSF across diverse clinical contexts [25]. For HHV-6 in particular, qualitative CSF detection may occur with active infection, reactivation, incidental detection or iciHHV-6 [13–15, 23, 26]. However, in the absence of linked clinical and quantitative evidence, the predominance of HHV-6 among the longer-interval pairs cannot distinguish among these possibilities.

### Target-specific patterns and biological interpretation

All seven recurrent VZV pairs had intervals <30 days, whereas the two recurrent HSV-2 pairs had first-to-last spans of 615 and 797 days, respectively. The 615 day trajectory included four positive dates, with consecutive gaps of 28, 226 and 361 days (Table S5). Clinically adjudicated recurrent lymphocytic meningitis cohorts have shown that HSV can be repeatedly PCR positive across separate disease episodes [19], but the two HSV-2 pairs in this study lacked symptom, treatment and episode-boundary data. They, therefore, cannot be classified as recurrent lymphocytic meningitis on interval and qualitative PCR alone. Likewise, the VZV pairs cannot be confirmed as clustered sampling within a single episode. These small counts should not be used to estimate recurrence risk or a characteristic interval for either virus.

HHV-6 requires caution because latency, reactivation and chromosomal integration can each complicate the clinical significance of a positive result. Qualitative CSF PCR cannot distinguish between these states [14, 15, 23, 26]. HHV-6 can integrate into telomeric regions [27]; the integrated form can be inherited [28, 29] and quantitative or droplet digital PCR performed on an appropriate cellular specimen can support identification of chromosomal integration [8, 9]. Such confirmatory testing was not available in this study. The 10 HHV-6 pairs separated by ≥30 days should, therefore, be considered a descriptive subgroup rather than suspected or confirmed iciHHV-6. Their restriction to the pre-implementation period also prevents treating the longer HHV-6 intervals as a reproducible target-specific feature.

### Clinical and laboratory implications

Inter-positive interval may provide contextual laboratory history, but the present data do not support a fixed decision threshold. Recurrent qualitative PCR results should be interpreted in light of the viral target, prior testing history and clinical course rather than taken alone as evidence of new or ongoing CNS infection. When HHV-6 is repeatedly detected, and clinical interpretation remains uncertain, quantitative or integration-focused testing may be considered if clinically indicated and locally available.

Clinical variables that would be important for patient-level interpretation – including antiviral exposure, treatment changes, adherence, resistance, symptoms, care setting, the indication for repeat testing and ordering-clinician identity – were not available in the de-identified LIS dataset. These data would be required to determine whether repeat testing occurred within continued evaluation, whether antiviral treatment influenced subsequent detection, or whether repeated positivity represented one episode or biologically distinct events. Their absence limits clinical adjudication and precludes post-hoc mechanistic classification.

### Implementation-period sensitivity

The count-only comparison showed 24 recurrent pairs in the pre-implementation calendar period and eight in the post-implementation period, but the periods differed substantially in duration and testing volume. Expressed per 1,000 interpretable target-level results, the crude recurrent-pair count was 1.83 before and 1.17 after implementation. Using positive patient–virus pairs as the denominator, within-period recurrent positivity was 24/771 (3.1%) and 8/176 (4.5%), respectively. Restricting the denominator to positive pairs with subsequent same-target testing yielded 24/57 (42.1%) and 8/23 (34.8%). These conditional proportions describe selected, non-comparable retested subgroups; neither they nor the all-positive-pair proportions estimate clinical recurrence risk.

Seventeen of the eighteen recurrent HHV-6 trajectories occurred in the pre-implementation calendar period. HHV-6 target-level positivity also differed between periods (9.9% pre-implementation versus 5.5% post-implementation). The aggregate HHV-6 pattern was, therefore, derived largely from the earlier period and may reflect period-specific differences in assay mix or workflow, ordering practice, case mix or follow-up rather than a stable biological feature of recurrent HHV-6 positivity.

Calendar period was not equivalent to assay platform, and row-level platform identity, ordering context, case mix and follow-up processes were unavailable. The observed HHV-6 concentration, therefore, cannot be attributed specifically to Seeplex, FilmArray, ordering practice, population differences or follow-up. Unequal follow-up opportunity and calendar-period censoring also limit interpretation of the proportions. This analysis demonstrates neither a Seeplex-related artefact nor analytical equivalence between platforms, and the aggregate HHV-6 pattern may not generalize to the post-implementation workflow.

### Strengths and limitations

Strengths include the 16 year observation period, preservation of longitudinal patient identifiers and test dates, patient–virus pair-level analysis and sensitivity analyses across four operational interval thresholds. Supplementary validation documentation records the analytic definitions and aggregate checks used to verify the recurrent-pair counts and exploratory Fisher’s exact analyses.

This study has several important limitations. First, only 33 recurrent patient–virus pairs were identified, limiting precision and precluding robust target-specific inference. Only 492/4,272 patients (11.5%) underwent testing on two or more dates; the recurrent cohort, therefore, arose from a selectively retested subgroup. Although 33/1,934 same-target retested patient–virus pairs (1.7%) met the recurrent-positivity definition, this proportion is a laboratory-observation frequency and not a patient-level disease-recurrence risk. Second, the 30-day threshold was operational rather than biologically validated, and admission-level episode boundaries were unavailable. Third, eight pairs had more than two positive dates. Their consecutive-positive intervals are provided in Table S5, but the first-to-last span can still obscure shorter gaps, and neither measure identifies clinical episode boundaries. Fourth, the analysis unit was the patient–virus pair; the 33 pairs represented 33 unique patients, and no patient contributed more than one recurrent viral target. Thus, the pair-level analysis did not include clustering of multiple recurrent viral targets within the same patient. Fifth, target availability and testing frequency were not uniform across viruses or calendar periods, particularly for CMV. Sixth, the dataset was not comprehensively linked to care setting, ordering clinician or indication, symptoms, final diagnosis, immune status, CSF indices, neuroimaging, antiviral therapy, adherence, resistance, Ct values or quantitative viral loads. Consequently, recurrent pairs could not be distributed among persistence, reactivation, recurrent infection or iciHHV-6 categories. Finally, the testing workflow changed over time. The denominator-aware comparison reduces distortion from unequal period size but remains susceptible to unequal retesting opportunity, follow-up and calendar-period censoring; it is a temporal sensitivity analysis rather than a direct assay-platform comparison.

## Conclusion

Across 16 years of CSF herpesvirus PCR testing, 33 recurrent patient–virus pairs showed heterogeneous target composition and inter-positive intervals. HHV-6 contributed most pairs separated by ≥30 days, all from the pre-implementation period; calendar-period differences prevent attributing this pattern to the viral target alone. The primary exploratory comparison was not statistically significant and the interval thresholds were operational. These data do not distinguish clustered repeat testing, persistent DNA detection, reactivation, recurrent infection or chromosomal integration. Recurrent qualitative CSF PCR results should, therefore, be interpreted in conjunction with prior testing history and, where clinically relevant, linked clinical and quantitative virological data. Multicentre studies with clinical adjudication are needed to determine whether inter-positive interval patterns have reproducible diagnostic or laboratory-stewardship value.

## Supplementary material

10.1099/jmm.0.002222 Supplementary Material 1.

## Abbreviations

CMV: cytomegalovirus
CNS: central nervous system
CSF: cerebrospinal fluid
Ct: cycle-threshold
EBV: Epstein–Barr virus
HHV-6: human herpesvirus 6
HSV-1: herpes simplex virus type 1
HSV-2: herpes simplex virus type 2
iciHHV-6: inherited chromosomally integrated HHV-6
IQR: interquartile range
LIS: laboratory information system
ME: meningitis/encephalitis
VZV: varicella-zoster virus
