# Supplementary material for: Recurrent cerebrospinal fluid herpesvirus PCR positivity: a retrospective analysis of inter-positive intervals
Source: J Med Microbiol. 2026 Nov 11;75(11):002222. doi: 10.1099/jmm.0.002222 (PMC13754281; doi:10.1099/jmm.0.002222)
Supplement: Supplementary file 1 [file jmm-75-02222-s001.pdf]

Supplementary Material

Recurrent cerebrospinal fluid herpesvirus PCR positivity: a retrospective analysis of inter-positive intervals

Supplementary Table S1a

Target-specific classification across operational inter-positive interval cut-offs.

Cut-off (days)	Target	Recurrent pairs	Shorter interval	Longer interval
14	HHV-6	18	6	12
14	VZV	7	7	0
14	HSV-1	5	3	2
14	HSV-2	2	0	2
14	CMV	1	1	0
14	Total	33	17	16
30	HHV-6	18	8	10
30	VZV	7	7	0
30	HSV-1	5	3	2
30	HSV-2	2	0	2
30	CMV	1	1	0
30	Total	33	19	14
60	HHV-6	18	8	10
60	VZV	7	7	0
60	HSV-1	5	4	1
60	HSV-2	2	0	2
60	CMV	1	1	0
60	Total	33	20	13
90	HHV-6	18	10	8
90	VZV	7	7	0
90	HSV-1	5	4	1
90	HSV-2	2	0	2
90	CMV	1	1	0
90	Total	33	22	11

Shorter: below the stated cut-off. Longer: at or above the cut-off. These are operational categories, not validated biological episode boundaries.

Supplementary Table S1b

Exploratory comparison of longer intervals between HHV-6 and non-HHV-6 targets.

Cut-off (days)	HHV-6 longer / total	Non-HHV-6 longer / total	HHV-6 share of longer pairs	Fisher P
14	12/18	4/15	75.0%	0.037
30	10/18	4/15	71.4%	0.158
60	10/18	3/15	76.9%	0.072
90	8/18	3/15	72.7%	0.266

P-values are two-sided, exploratory and unadjusted for multiple testing. The primary 30-day comparison yielded P = 0.158. The isolated nominal P = 0.037 at 14 days does not provide confirmatory evidence of a target association.

Supplementary Table S2

Continuous first-to-last inter-positive interval summaries, in days.

Target	Pairs, n	Median	IQR (Q1–Q3)	Range
All targets	33	11	4–185	1–864
HHV-6	18	61.5	6.5–247.25	2–634
VZV	7	5	1–7.5	1–10
HSV-1	5	11	3–36	3–864
HSV-2	2	706	660.5–751.5	615–797
CMV	1	6	6–6	6–6

IQR, interquartile range. Quartiles use linear interpolation (Hyndman–Fan type 7). For CMV (n = 1), the displayed quartiles and range equal the single observed interval. Target-specific summaries describe these observed pairs and should not be interpreted as typical disease-recurrence intervals.

Each interval is the number of days from the first to the last positive order date for one recurrent patient–virus pair after same-day consolidation. It is not a consecutive-positive gap when a trajectory contains more than two positive dates.

Supplementary Figure S1

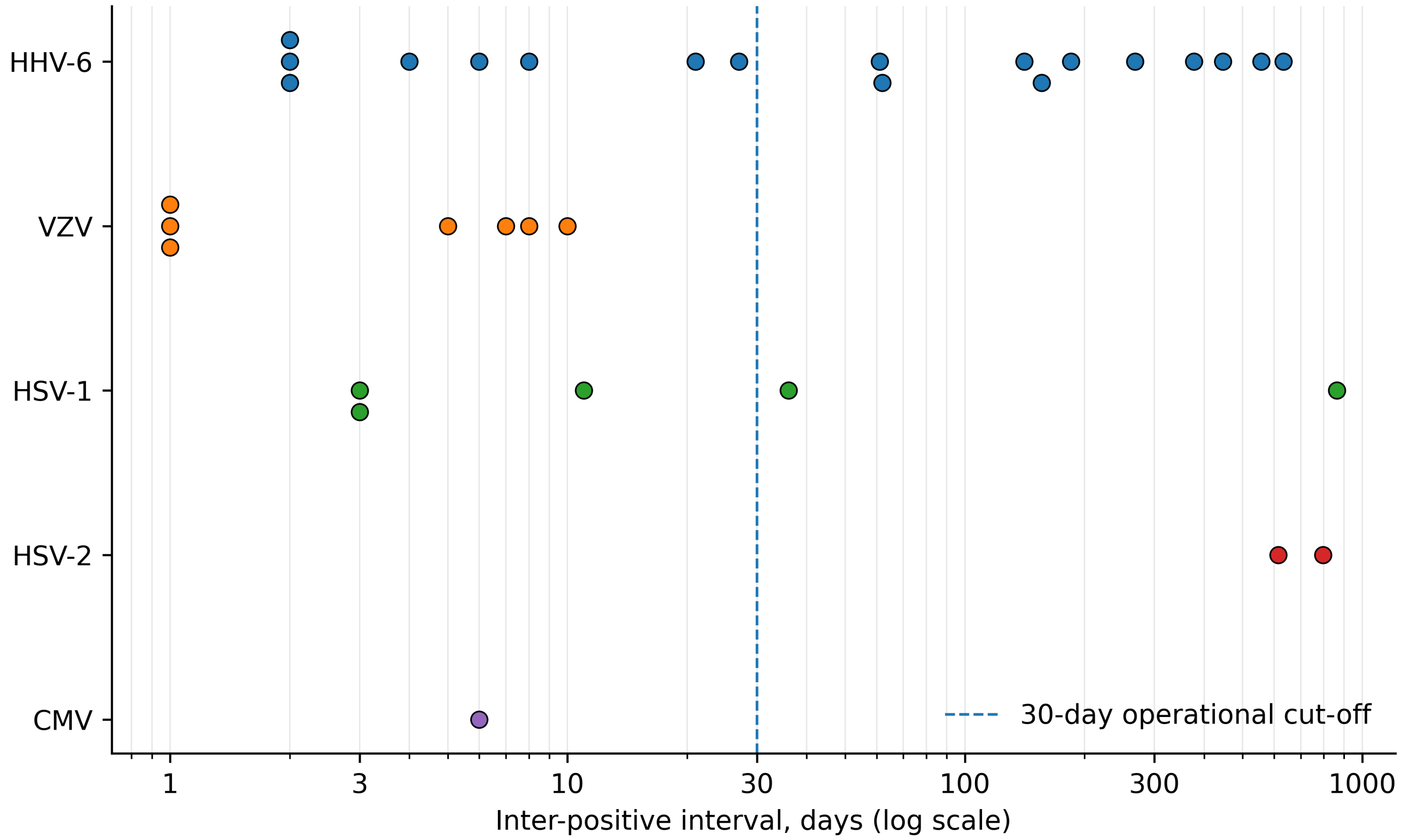

First-to-last inter-positive intervals for all 33 recurrent patient–virus pairs, grouped by viral target. Each dot represents one observed interval. The horizontal axis uses a logarithmic day scale; vertical offsets separate the dots and have no clinical meaning. The dashed line marks the operational 30-day cut-off. Overall median: 11 days (IQR, 4–185; range, 1–864).

All 10 HHV-6 pairs with intervals ≥ 30 days occurred in the pre-implementation period; calendar-period differences limit target-specific interpretation.

CMV, cytomegalovirus; HHV-6, human herpesvirus 6; HSV-1, herpes simplex virus type 1; HSV-2, herpes simplex virus type 2; VZV, varicella-zoster virus.

Alt text: Strip plot of first-to-last intervals for 33 recurrent patient–virus pairs by viral target on a logarithmic day axis. The median is 11 days and the range is 1–864 days. A dashed line marks 30 days.

Supplementary Table S3

Recurrent trajectories by implementation period and 30-day interval class.

Period	Pairs	<30 days	≥30 days	HHV-6
Pre	24	12	12	17
Post	8	7	1	1
Transition-spanning	1	0	1	0

Supplementary Table S4

Implementation-period denominators and observed subsequent same-target testing.

Measure	Pre	Post
Interpretable patient–virus–date results	13,091	6,765
Positive patient–virus pairs	771	176
Positive pairs with a later same-target test, n/N (%)	57/771 (7.4)	23/176 (13.1)
Pairs with ≥2 positive dates, n/N positive pairs (%)	24/771 (3.1)	8/176 (4.5)
Recurrent-positive among subsequently retested positive pairs, n/N (%)	24/57 (42.1)	8/23 (34.8)
HHV-6-positive pairs	302	84
HHV-6-positive pairs with a later test	31	6
HHV-6 pairs with ≥2 positive dates	17	1

Pre: 16 March 2010 to 9 November 2020. Post: 10 November 2020 to 30 March 2026. Calendar periods are not mutually exclusive assay-platform groups. Same-day records were consolidated using a positive-wins rule. Subsequent retesting requires a later same-target result after a positive date within the same period.

The transition-spanning HSV-2 pair had one positive date per period. It contributes to both positive-pair denominators but neither within-period recurrent numerator (24 + 8 + 1 = 33 trajectories). Period denominators are not additive. The 42.1% and 34.8% proportions describe selectively retested subgroups and are not recurrence-risk estimates. Observation duration, testing practices and follow-up opportunity differ.

Analytic definitions and verification

Validation measure	Recalculated result
Interpretable target-level results / positive results	19,959 / 994
Patients / testing episodes	4,272 / 4,963
Patients tested on ≥2 dates	492 / 4,272 (11.5%)
Recurrent pairs / same-target retested pairs	33 / 1,934 (1.7%)
Recurrent pairs / unique recurrent patients	33 / 33
Raw results / positives, pre	13,149 / 809 (6.2%)
Raw results / positives, post	6,810 / 185 (2.7%)
HHV-6 raw results / positives, pre	3,329 / 329 (9.9%)
HHV-6 raw results / positives, post	1,559 / 85 (5.5%)
Pairs with more than two positive dates	8 / 33 (24.2%)

Analytic definitions

Interpretable binary results for HHV-6, VZV, HSV-1, HSV-2 and CMV were included from 16 March 2010 to 30 March 2026. A testing episode is a unique patient and order date. Same-day patient–virus records were consolidated using a positive-wins rule. Recurrent positivity requires at least two distinct positive dates. First-to-last spans are the primary intervals. Consecutive gaps are differences between adjacent positive dates, not intervals between all successive tests; intervening negative tests do not alter this definition.

Verification

The 33 first-to-last spans and consecutive-positive gap sequences were reconstructed from the longitudinal laboratory records. Recurrent-pair counts, period-specific denominators and two-sided Fisher exact P-values were recalculated using the stated definitions and agreed with the reported results. At cut-offs of 14, 30, 60 and 90 days, P-values were 0.037, 0.158, 0.072 and 0.266, respectively. These unadjusted exploratory comparisons depend on the cut-off; the isolated nominal $P = 0.037$ does not provide confirmatory evidence of a target association.

Source patient identifiers and calendar dates are omitted. Table S5 uses table-specific labels and reports interval values only. These trajectories describe laboratory detection, not adjudicated clinical recurrence.

Supplementary Table S5

Consecutive-positive intervals for recurrent patient–virus pairs with more than two distinct positive dates.

Study label	Target	Positive dates, n	First-to-last span, days	Consecutive gaps, days
S5-01	HHV-6	3	2	1, 1
S5-02	HHV-6	3	6	1, 5
S5-03	HHV-6	4	21	4, 5, 12
S5-04	HHV-6	3	377	255, 122
S5-05	HHV-6	7	557	31, 7, 28, 351, 113, 27
S5-06	HSV-1	4	11	1, 7, 3
S5-07	HSV-1	3	36	27, 9
S5-08	HSV-2	4	615	28, 226, 361

Eight of 33 recurrent pairs (24.2%) had more than two positive dates: five HHV-6, two HSV-1 and one HSV-2. Four had three dates, three had four dates and one had seven dates. Each remaining pair had two positive dates, for which the consecutive gap equals the first-to-last span.

Gaps are ordered chronologically and sum to the first-to-last span for each row. Same-day duplicates count as one positive date under the positive-wins rule. Study labels were assigned solely for this table and are not source patient identifiers. The first-to-last analysis was retained; gaps were not treated as independent observations in new hypothesis tests.

HHV-6, human herpesvirus 6; HSV-1 and HSV-2, herpes simplex virus types 1 and 2. First-to-last and consecutive intervals alone do not distinguish persistent detection, reactivation or separate clinical episodes.
